# Older patients’ attitudes towards and experiences of patient-physician end-of-life communication: a secondary analysis of interviews from British, Dutch and Belgian patients

**DOI:** 10.1186/1472-684X-11-24

**Published:** 2012-11-27

**Authors:** Natalie Evans, H Roeline W Pasman, Sheila A Payne, Jane Seymour, Sabine Pleschberger, Reginald Deschepper, Bregje D Onwuteaka-Philipsen

**Affiliations:** 1 VU University Medical Center, EMGO+ Insitute, Van der Boechorststraat 7, 1081, Amsterdam, BT, The Netherlands; 2 Lancaster University, Lancaster, UK; 3 University of Nottingham, Nottingham, UK; 4 UMIT-The Health and Life Sciences University, Hall & Vienna, Vienna, Austria; 5 VU University Brussels, Brussels, Belgium

**Keywords:** Palliative, End-of-life, Communication, Decision-making, Older patients, Qualitative

## Abstract

**Background:**

Older patients often experience sub-standard communication in the palliative phase of illness. Due to the importance of good communication in patient-centred end-of-life care, it is essential to understand the factors which influence older patients’ communication with physicians. This study examines older patients’ attitudes towards, and experiences of, patient-physician end-of-life (EoL) communication in three European countries.

**Methods:**

A secondary analysis of interviews from British, Dutch and Belgian patients over the age of 60 with a progressive terminal illness was conducted. Cross-cutting themes were identified using a thematic approach.

**Results:**

Themes from 30 interviews (Male n = 20, Median age 78.5) included: confidence and trust; disclosure and awareness; and participation in decision-making. Confidence and trust were reinforced by physicians’ availability, time and genuine attention and hindered by misdiagnoses and poor communication style. Most participants preferred full disclosure, though some remained deliberately ill-informed to avoid distress. Patients expressed a variety of preferences for and experiences of involvement in medical EoL decision-making and a few complained that information was only provided about the physician's preferred treatment.

**Conclusions:**

A variety of experiences and attitudes regarding disclosure and participation in decision-making were reported from each country, suggesting that communication preferences are highly individual. It is important that physicians are sensitive to this diversity and avoid stereotyping. In regard to communication style, physicians are advised to provide clear explanations, avoid jargon, and continually check understanding. Both the ‘informed’ and the ‘shared’ patient-physician decision-making models assume patients make rational choices based on a clear understanding of treatment options. This idealized situation was often not reflected in patients’ experiences.

## Background

The majority of deaths in Europe occur over the age of 65. However, the needs of older patients are often neglected during end-of-life (EoL) care [1]. Older patients have less access to specialist palliative care services than younger patients and are more likely to have their treatment needs under-assessed [1,2]. Patients and carers frequently identify communication as one of the most important aspects of good EoL care [3,4]. There is however evidence that older patients are given less time during the physician-patient interview [5], are provided less information on their diagnoses and prognoses [2,6] and are less likely to discuss their EoL preferences [2,7].

Sub-standard communication is both a cause and an outcome of poor EoL care. Poor communication affects physicians’ ability to recognize patients’ palliative care needs and make referrals to specialist services [2]. Furthermore, poor patient-physician communication influences patients’ understanding of their advanced condition, participation in treatment decisions and satisfaction with services [3,4,8]. These issues might be pertinent for all patients however they are particularly relevant for older patients whose characteristics suggest specific communication needs. For example, generational differences in communication style mean that older patients may afford physicians more respect and be less likely to question their judgments than other patient groups [5]. Older patients also suffer more multiple morbidities, leading to greater health care needs [1], and are more likely to suffer from impaired cognitive functioning.

Due to the importance of good patient-physician communication in EoL care, it is essential to understand the factors that influence communication between older patients and physicians. European surveys that explore patient-physician communication and participation in decision-making at the EoL have revealed that country of residence is a strong determinant of practices and attitudes [9-12]. For instance, surveys mapping physicians’ actual and intended discussion with patients on a number of EoL issues have found significant cross-country differences, such as more frequent discussions in the Netherlands and less frequent discussions in Italy than other European countries [9-11] (the United Kingdom (UK) was not included in these surveys). These between country differences were attributed to cultural factors [9-11]. Culture, a term which has been variously defined but which the authors understand as ‘a system of shared ideas and meanings that underlie, influence and structure the ways in which people think and act in practical situations’ [13], influences both the content and the process of patient-physician communication. Culture shapes patients’ and physicians’ expectations concerning accepted patterns of communication, defined roles of the physician and the patient and topics discussed [14].

Although surveys are excellent for mapping differences in practices and attitudes across Europe, a deeper understanding of patients’ and physicians’ experiences, attitudes and preferences surrounding EoL communication is most readily achieved through qualitative rather than quantitative research [15,16]. Unfortunately, the growth in cross-country quantitative EoL research in Europe in recent years has not been accompanied by a comparable increase in qualitative research. This paucity is undoubtedly influenced by the relatively high-cost and time-consuming nature of qualitative approaches [17], as well as the challenges of multi-lingual and trans-cultural research [18,19]. Since cross-country qualitative research is still in its infancy there is currently no study available with a specific focus on EoL communication issues comparing qualitative data from different European countries.

By reporting the results of an exploratory secondary analysis of older patients’ interviews from three northern European countries, this paper begins to address the paucity of cross-country qualitative research on EoL communication in Europe. The interviews analyzed were conducted as part of studies that explored terminally ill patients’ attitudes towards, and experiences of, death, dying and EoL care. As the primary studies varied in their specific foci, this cross-country comparison only examines common themes surrounding patient-physician EoL communication. In light of calls to implement European-wide EoL care policies and for best practice models to be used across Europe [1,20], such an approach assists the identification of common issues in patient-physician EoL communication throughout Europe and potentially contributes to the formulation of Europe-wide policy recommendations.

This study therefore aims to examine older patients’ attitudes towards, and experiences of, patient-physician EoL communication in the United Kingdom, the Netherlands and Belgium. Specific objectives include: 1) to examine common themes surrounding patient-physician EoL communication from British, Dutch and Belgian interviews with older patients on attitudes to death, dying and EoL care; and 2) to identify similarities and differences in attitudes and experiences within these common themes.

## Methods

### Secondary analysis

#### Selection of studies and participant interviews

This study is part of a two part project. The first part consisted of a detailed reflection on ethical and methodological challenges in interviewing older adults about EoL issues, comparing experiences from six qualitative studies in four European countries [21]. This article however focuses more narrowly on older patient-physician EoL communication. As such it was necessary to assess the nature of the studies and the quality of the interviews included in the methodological reflection, to ensure the phenomenon of interest was sufficiently represented and the interviews and related documents were complete [22,23]. Interviews from one of the studies were no longer available [24]. A number of transcripts (minimum three) and associated field notes from each of the remaining five studies were critically reviewed by the first and second authors (NE and RP). Two studies were excluded as they either did not cover patient-physician communication [25] or did not involve a palliative care population [26]. Interviews from the remaining studies were deemed to provide sufficient depth and detail concerning older patients’ experiences of, and attitudes towards, patient-physician EoL communication.

This secondary analysis therefore draws upon data from these three qualitative studies conducted in the United Kingdom, the Netherlands and Belgium (Table 1). Participants from all studies had been diagnosed with a progressive terminal illness and were receiving EoL care. Whilst the primary aims of the studies ranged from exploring patient-GP EoL communication [27], identifying aspects of EoL care valued by the patient [28] and understanding cultural constructs of loss, transition and adaptation, all the interviews were largely unstructured and aimed at generating patients’ narratives [21] (Table 1). The interviews from all studies had been transcribed verbatim for the purposes of conducting thematic analysis. Details of the primary studies’ aims, informed consent, and funding sources can be found in Table 1.

**Table 1:** Details of primary studies

**Country**	**Primary study title**	**Aim**	**Participants**	**Patient consent**	**Funding**
**Belgium**	Medical and ethical quality of care when taking EoL decisions.	To develop a guideline for general practitioners (GPs) on EoL communication with patients who wish to die at home.	Patients with terminal illnesses (n = 17).	Informed consent was obtained verbally.	Belgian Science Policy.
**The Netherlands**	End-of-life care in general practice in the Netherlands.	To explore the aspects valued by both patients and GPs in EoL care at home.	Patients with terminal illnesses in the care of a GP (n = 30).	Written informed consent obtained.	Centre for Development of Palliative Care Amsterdam, and the Ministry of Health, Welfare and Sports.
**United Kingdom**	Ethnicity and cancer: examining psychosocial transitions for older people.	To investigate the cultural constructs of loss, transition, and adaptation when encountering a diagnosis of a life threatening illness; to elicit narratives from older adults about their experiences of cancer diagnosis.	Chinese (n = 24) and white (n = 47) hospice day centre patients.	Written informed consent obtained.	Dimbleby Cancer Care.

A sub-sample of available interviews was purposively selected from each study: participants over the age of 60 with a range of socio-demographic characteristics, health statuses and care locations were included. One of the studies (Ethnicity and Cancer) compared the views of older Chinese and white patients resident in the UK. From this study Chinese participants (n = 24) were excluded, leaving only white older patients (n = 47), in order to make the sample’s ethnic composition comparable to the samples from the Netherlands and Belgium. A final sub-sample of 30 interviews (United Kingdom = 10, the Netherlands = 11, Belgium = 9) was selected from 94 interviews (United Kingdom = 47, the Netherlands = 30, Belgium = 17).

#### Thematic analysis

The selection and construction of data are intertwined with the assumptions and procedures of the intended analytical approach [29]. Thematic approaches had been used to identify key themes in all three included studies. Thematic analysis was also, therefore, appropriate for the secondary analysis and key cross-cutting themes were identified using a constant comparison approach [30].

After a reading of the English language interview transcripts by the first author (NE), and of the Dutch and Flemish interview transcripts by the second author (RP), a preliminary coding scheme was developed. Both researchers used this coding scheme to code all interviews. Interview segments from the non-English language transcripts that dealt with patient-physician interactions were identified by the second author (RP) and translated into English by professional translators. The language expertise provided by the professional translators, combined with the authors’ language skills and understanding of the research topic enabled a sensitive and nuanced translation of the interview data [18]. The preliminary coding scheme also provided the basis for an iterative process of coding and identification of emergent themes conducted by the first author (NE) using the full English language transcripts and the translated sections of the non-English language transcripts. Codes and emergent categories were compared and contrasted until a number of key cross-cutting themes were identified from the data [30]. Interview transcripts and field notes were managed and coded using Atlas ti qualitative data analysis software [31].

#### Ethics approval and informed consent

Ethics approval was obtained for all of the primary studies (Table 1). Either verbal or written informed consent was gained from all primary study participants. The conditions of the primary studies’ ethics approval were reviewed to ensure that secondary analysis of interview data was in line with the aims of the informed consent obtained from the participants [25]. Furthermore, the researchers involved in the primary research thoroughly anonymized the data [32].

#### Theoretical considerations and methodological issues

Although the secondary analysis of datasets is most readily associated with quantitative research, there is increasing interest in the secondary analysis of qualitative data [32]. There are a number of theoretical and methodological issues involved in applying such approaches to qualitative data. Quantitative research is often described as being based on a positivist paradigm whereas qualitative research is based on a constructivist paradigm [33]. Although this dichotomy is somewhat simplistic [34], it is useful for understanding the epistemological issues involved in the secondary analysis of qualitative data. Within a constructivist paradigm reality is created between the observer and the observed and qualitative inquiry does not aim to reveal a single objective, measurable reality [33]. As such the context in which qualitative data are created is central to their interpretation. The secondary analysis of qualitative data is often criticized because this vital understanding of context is lost [35].

Van den Berg [29] however argues that secondary analysis of qualitative data is feasible when the contextual information most relevant for the interpretation of the text is provided. Attempts to maintain sufficient contextual information for the current secondary analysis are described in Table 2. Furthermore, measures taken to ensure the rigour of the secondary analysis, as recommended by Heaton [36] and Van den Berg [29], are detailed in Table 2.

**Table 2:** Recommendations for the reporting of secondary analyses of qualitative data and measures taken

**Recommendation to ensure rigour in secondary analysis of qualitative data**	**Measure taken**
Information about the discursive context of interviewee's responses.	Detailed transcriptions, transcribed verbatim, were available.
Information about the discursive history of interviewee's responses.	Whole interviews were available to at least one of the researchers involved in the secondary analysis rather than just the relevant sections.
Information about background characteristics of interviewer and interviewee.	Field note summaries (interview ‘pen portraits’), included information about the participant’s gender, age, socio-economic status, residence, family situation, key life events and the context in which each interview took place were provided.
Information about the place, time and setting of the interview, such as presence of third persons.	Information included in detailed field note summaries.
Information about the composition of the secondary dataset.	Sampling frameworks of the primary studies and the selection of the subset used in the secondary analysis are described in the methods section.
Funding of the primary and secondary work.	Funding information for the primary studies is included in Table 1. Additional funding sources for the secondary analysis are included in the acknowledgements.
The relationship of each of the authors to the data.	At least one researcher from the original study was available to provide further information on any contextual queries (ShP, BP, RD). The original researchers were not however involved in the re-coding of the data (conducted by NE and RP).
Information about informed consent.	Ethics and informed consent are described in the methods section.
Rationale for approach used in secondary analysis and a description of analysis procedure.	Rationale for the thematic analysis is described in the methods section.
Information about how the data were managed.	Data were managed using Atlas ti qualitative data analysis software.
Information about how the rigour of the analysis was established.	Details provided in this table.
Information about how the ‘fit’ of the data was ascertained.	At least one researcher from the original study confirmed the fit of the resulting themes.
Details of limitations.	Limitations are outlined in the limitations section.

## Results

The majority of participants had cancer, whereas just under a quarter suffered from non-malignant conditions (Table 3). The median age was 78.5 and the majority of participants were male (67%) (Table 3).

**Table 3:** Characteristics of participants included in the purposive sub-sample

		**Belgium**	**The Netherlands**	**The United Kingdom**	**Total**	
		**Medical and ethical quality of care when talking end-of-life decisions**	**End-of-life care in general practice in the Netherlands**	**Ethnicity and cancer**		
		**n = 9**	**n = 11**	**n = 10**	**n = 30**	
		**n**	**n**	**n**	**n**	**%**
Age	60 - 64	2	0	1	3	10
	65 - 74	5	1	1	7	23
	75 - 84	1	8	6	15	50
	85>	1	2	2	5	17
Sex	male	7	7	6	20	67
	female	2	4	4	10	33
Condition	Cancer	9	5	9	23	77
	Non-cancer	0	6	1	7	23

The analysis provided insight into the nature of the patient-physician interaction for older patients receiving EoL care. Cross-cutting themes included: confidence and trust; disclosure and awareness; and participation in decision-making.

### Confidence and trust

The physician-patient encounter is affected by patients’ expectations, which are shaped in part by their past experiences with physicians and other healthcare professionals. Factors that reinforced older patients’ trust and confidence in their physician included availability, time and genuine concern.

**Participant’s wife:** But if we call him, he’s there for us. It’s true. He doesn’t count it. He says, “I’m getting up and I’m there,” and ten minutes later there he is. He doesn’t live far away, but he still has to come here.

Participant 8, Male, 65 – 74 years, Belgium

**Interviewer (I):** Are there any things regarding the doctor about which you are less satisfied?

**Participant (P):** No, not at all. Nothing with regard to the general practitioner or with regard to the lung specialist. That’s a really nice woman. I can call her at any time of the day. Even in the middle of the night, at her home number!

Participant 10, Male, 75 – 84 years, the Netherlands

**P:** […] I think in general principle, this place [the hospice] has been superb. The ordinary hospital sort of stuff that you get is in and out, flash, bang, wallop and really has nothing, there's nothing humane about it whatsoever.

Participant 21, Male, 75 – 84 years, United Kingdom

Participants frequently commented on their physician’s availability, the amount of time the physician dedicated to them and if the physician appeared to be in a hurry. Furthermore, older patients emphasized how important it was that their physician gave them their full attention and, even if they had only a little time to spare, that their concern was genuine.

Negative experiences also greatly impacted older patients’ trust and confidence in their physician. Some participants specifically criticized the manner in which physicians spoke to them, describing a lack of empathy and sensitivity. Whereas others criticized physicians’ use of medical jargon and expressed satisfaction with physicians who were prepared to explain difficult to understand medical terms.

**I:** [ …] did you still trust that general practitioner?

**P:** No, I had asked for a walker then. And then he said, 'that’s not worth the trouble anymore.'

Participant 12, Male, 75 – 84 years, the Netherlands.

**P:** I said "he’s [the physician] a Canadian and he's as cold as a bloody fish that they pull out of the sea in Canada" […] So he is a cold fish and I mean that sincerely, but what does he deal with? Death constantly. And all he's ever got to say to some poor bugger is "I'm afraid that you're on the way out". […] [b]ut I do wish that he'd sort of look at me as if to say "well, I do appreciate how you feel", but I understand how he has to cope with it and that's it. So we beg to differ at times, you know. If he says something I don't agree with, I'll bloody tell him so. Because I've got nothing to lose, have I?

Participant 21, Male, 75 – 84 years, United Kingdom

**P:** But you’d prefer a doctor to speak to you as a human being, and not use all these formal terms.

**I:** They use too many formal words?

**P:** Yes, when my daughter was here too, she even said so to the specialist.

Participant 5, Male, 65 – 74 years, Belgium

Older patients’ confidence in physicians and the efficacy of biomedicine were also negatively affected by experiences of misdiagnoses and inappropriate treatments.

**I:** Yes, you were on the wrong track really weren't you [in reference to a misdiagnosis by Dr. Smith]?

[…] So that's the difficult one is the fact it wasn't picked up;

**P:** That's the difficult one yes.

Participant 22, Male, 60 – 64 years, United Kingdom

### Disclosure and awareness

Older patients frequently expressed a preference for open and honest disclosure of diagnoses and prognoses. Indeed, the one woman who knew that she had not received a prompt disclosure of her diagnosis stated that she would have preferred full disclosure.

**P:** My GP, when he’d got the letter [from the specialist]…He said, “Look, Denise, I’ve had this letter a while. I’ll give it to you now,” he said, “but Denise, I should have told you in January; that you needed another scan… but it would’ve been the same. We couldn’t do anything about it anyway.” And he said that my morale was still so good that he didn’t want to… because that’s what he said, this doctor, that you can’t do anything about it. If I’d been three months earlier, then yes. And so I asked my GP why he hadn’t done anything with the letter for so long…

**I:** And you think your doctor should tell you?

**P:** (hesitantly) Yes, I do think so… although to be honest, Dr R. did do it in a gentle way.

Participant 1, Female, 60 – 64 years, Belgium

Patients also expressed dissatisfaction with the amount of information provided by their physician about their illness and treatment options. A common complaint was that information was something that needed to be sought and was not routinely provided.

In contrast, other patients preferred to remain deliberately unaware about their illness and treatment options in an attempt to avoid distress.

**I:** Did they [the GP] discuss it with you?

**P:** Not much. Those doctors don’t say much.

I’d prefer not to know. Otherwise it breaks my heart.

**I:** Did they tell you what to expect?

**P:** No. I prefer not to know; it would make me sick.

I’ve already had a fever for three days. I don’t want to know.

**I:** Have you talked about what you want to say or decide?

**P:** There’s no point in asking me anything, I can’t answer you. Unfortunately. I don’t know…

**I:** Is there a doctor who has explained things when you’ve asked anything?

**P:** No. They don’t say anything.

**I:** Have you ever asked anything or would you like to have asked anything?

**P:** No, if they don’t say anything, then I don’t ask. I don’t say anything. They definitely don’t say anything.

Participant 2, Female, 75 – 84 years, Belgium

**P:** Yes, well when I came here I saw the doctor the same day. And the doctor said, ‘Now I’m going to…’ I was in an hour. ‘Now I said when you’ve gone I shall be writing to your GP’, and he said, ‘you’ve got every right to ask to see the letter. Now what do you want to do?’ So I said, ‘I don’t. Whatever you say to him, I don’t want to read… hear about it you see.’

Participant 29, Female, 85 ≥ years, United Kingdom

When asked if a physician should provide full information about diagnosis and prognosis, some older patients were pragmatic and stated that a physician should know when and for whom full information is appropriate. Others stated that a physician should always be open and direct in communication.

An additional complaint was that, when information on treatments was given, the physician only provided information concerning the treatment option they recommended and did not discuss the consequences of other actions.

**P:** Yes convinced, he didn’t leave me any choice, they don’t leave you any choice. My reward is that I’m not given any choice […] So there were two possibilities: chemotherapy or not. And I already knew that chemotherapy could also have physical consequences and stuff, vomiting and feeling bad and everything. But what actually happened if I refused chemotherapy. I didn’t actually hear anything about that. So I think that was missing. So I didn’t actually make a decision, err, with any conviction. Yes. Correct me if I’m wrong.

Participant 6, Male, 65 – 74 years, Belgium

**P:** And I regret that. That they pushed me [to get started with a treatment]. You just grasp at a straw. So when they tell you: 'You are terminally ill; you have cancer and the only possibility is surgery' then you want to seize that possibility, right? However, afterwards, it turned out that it was so radical. When I had to go to the hospital and that they first told me that: first with that doctor, then with that internist, to the surgeon, and that only then I found out that it was way too radical […] Every time I make a phone call, I have to recover my breath. At those times, I’m jealous that I was not told about the possibility of being ‘taped’ [an alternative procedure] from the start [instead of surgery] […]Then I went to the lung specialist and I said: 'I changed my mind, I'm not going to do it'. And she said: 'I'm glad you didn't go through with it.' Because they push you to do it! [to become a study subject] However, they just need people. Because, in twenty years, there will be an explosion of asbestos victims who have all worked there.

Participant 10, Male, 75 – 84 years, the Netherlands

### Participation in decision-making

In each country older patients expressed a variety of preferences for and experiences of involvement in medical EoL decision making. Levels of involvement included: active involvement in decisions; involvement with family support; and no involvement in decisions. Many older patients described joint discussions about treatment options, including non-treatment decisions, with their physician.

**I:** Did you talk with the doctor about you not wanting euthanasia or resuscitation? Did this take place on your initiative or on the doctor's initiative?

**P:** Yes, on my own initiative. I’ve witnessed a resuscitation in the hospital and I thought it was horrible. And I do not want surgery either any more

Participant 12, Male, 75 - 84, the Netherlands

Some participants believed that patients should make decisions in collaboration with, or with the support of, their family and saw the physician as having more of an advisory role. The presence of a spouse or adult child at the physician-patient interview who provided emotional support or aided the understanding of options available was often mentioned.

**R**: […] I said, 'Would you mind if my son came. Can you explain everything to him?' 'Oh no that's a good idea.' So my son came and they told him what's what.

Participant 26, Male, 75 - 84, United Kingdom

**Wife:** No, no, it’s us who decides that [treatment], together with the specialist. But if you don’t want to, you don’t have to continue. They’d continue. They say it makes no difference anymore, but they want to continue anyway.

Participant 5, Male, 65 - 74, Belgium

Family involvement in decision-making was not however universally positive or supportive. Family members have their own hopes and expectations, which may differ from those of an older patient. The presence of family members was described by one participant as having a coercive effect on his decision to choose a more aggressive, experimental treatment, which he later regretted. 

**P:** All my children were there. So you are anxious all the time. Normally, you have a meeting with the general practitioner, but when all the children also have to be present, and they are going to make a decision and are all enthusiastic: "Dad, you have to get surgery' […] So, looking back, I actually regret this.

Participant 10, Male, 75 - 84, the Netherlands

In some cases the participant deferred decision-making responsibilities to the physician, whereas others were simply told what their treatment would be rather than being included in treatment (or non-treatment) decisions. This lack of involvement often went unchallenged and was treated fatalistically by participants.

**P:** So I was told in my case that they weren’t going to operate. And I accept that. OK? Yes, I can run from one hospital to the next and… err… to Aalst [Belgian city] or what’s it called?

Participant 6, Male, 65 - 74, Belgium

## Discussion

This exploratory secondary analysis of older patients’ interviews from three northern European countries highlights some common issues in patient-physician EoL communication. Furthermore, it represents the first step in addressing the paucity of qualitative cross-country research on patient-physician EoL communication in Europe. The identification of the common themes of ‘confidence and trust’, ‘disclosure and awareness’ and ‘participation in decision-making’ from Dutch, Belgian and British older patients’ interviews implies that they are relevant across diverse social and cultural settings.

Within each of these common themes, a diversity of participant experiences and attitudes were reported from each of the three countries. This suggests that attitudes and preferences of older people regarding EoL patient-physician communication are highly individual. The variety of preferences for information provision and decision-making (including the involvement of family members), revealed in the findings, highlights the lack of an ‘idealized’ preference for older patients. For example, although older patients frequently expressed a preference for open disclosure of their diagnosis and prognosis, others remained deliberately unaware by rejecting information offered by their physician. These patients can be considered in a state of self imposed ‘partial awareness’ of dying. Partial awareness, according the typology of Glaser and Strauss, includes the suspicion of dying or the pretence of not dying [37]. This finding underlines the importance of denial or partial knowledge as a coping mechanism for some patients. It is important that information should be frequently and sensitively offered, but never be forced upon such patients.

Whereas more quantitative approaches reveal that country of residence is a strong determinant of practices and attitudes [9-12], this qualitative secondary analysis emphasizes that, even though the proportions of people with a particular attitude or preference might vary between countries, each attitude or preference type is still represented in each country. Calls for cultural sensitivity in regard to communication in EoL care in Europe should therefore be interpreted as a need for a sensitive understanding and flexible inclusion of all preferences rather than the modification of recommendations and policy for different European countries [38]. It is not advisable to modify recommendations regarding participation in decision-making, disclosure and communication as such an approach runs the risk of reifying culture and neglects diversity in patient preferences. It is instead essential that physicians throughout Europe recognize the importance of ascertaining individual patients’ EoL communication preferences, which whilst influenced by culture are not determined by culture alone. Indeed culture is only one amongst a variety of factors that affect older patients’ EoL communication preferences. There remains a need to better understand the relations between different factors and how they depend on and influence each other.

Patients reported a variety of experiences of communication with physicians, ranging from caring to coercive. Trust and confidence were reinforced by physicians’ availability, time and genuine concern, and negatively impacted by previous negative experiences such as misdiagnoses and poor communication style (lack of sensitivity and empathy) in all three countries. These findings highlight the importance of continued EoL communication training for physicians.

The themes revealed by the secondary analysis are supported by the findings of international research on patient-physician EoL communication. Nolan et al. [39] described American patients’ diverse preferences for participation of physicians and family in decision making, whereas Pardon et al. [40] described similar results amongst Flemish patients. Aldred et al. [41] described older British heart failure patients’ dissatisfaction with physicians’ availability and information provision concerning their condition. Misunderstanding of some medical terms was also reported [41]. Heyland et al. [3] found that the majority of Canadian patients with advanced cancer considered trust and confidence in their physician as extremely important and that just under half considered the honest communication of information extremely important. It should be noted that all of the countries included in these studies, like those included in this secondary analysis, are from developed, high-income countries.

### Strengths and limitations

The studies, from which the interviews were derived, varied in their foci (though all explored older patients’ attitudes towards, and experiences of, death, dying and EoL care). As such the secondary analysis only looked at common themes on patient-physician EoL communication in three northern European countries. The variation found in the primary studies’ main foci does however make the identification of common themes even more remarkable.

Decontextualization is a major risk in secondary analysis of qualitative data. A number of steps, therefore, were taken to ensure sufficient contextual information was available to inform the analysis. These steps are outlined in Table 2.

### Further research

This secondary analysis only begins to address the paucity of cross-country qualitative research on patient-physician EoL communication in Europe. Furthermore, in Europe, EoL attitudes and practices are often discussed in terms of northern/southern, Protestant/Catholic, or Anglo-Saxon/Mediterranean dichotomies. However, all the interviews included in this secondary analysis come from northern European countries. Further research is required to ascertain whether the themes identified in this study are applicable in other European countries. There remains a need for primary cross-country research to better understand both the similarities and differences in attitudes, preferences, physicians’ practices and patients’ experiences across the continent, as well as the role of socio-economic characteristics and past experiences of illness, death or bereavement, to ensure the flexibility of patient care and the accommodation of a variety of patient preferences.

## Conclusion

This study highlights that older patients’ attitudes and preferences concerning patient-physician EoL communication are highly individual and that there is no ‘idealized’ preference for disclosure or participation in decision-making. Physicians must therefore be sensitive to a diversity of preferences amongst older patients and avoid stereotyping. In regard to communication style physicians are advised to be mindful to provide patients with clear explanations, avoid jargon, and continually check patients’ understanding. Physicians must also be careful to avoid partial information provision or any other attempt to manipulate older patients’ treatment choices as such actions can compromise patient autonomy and trust.

The findings have implications for the dominant patient-physician decision-making models. In both the ‘shared’ and the ‘informed’ decision-making models the doctor informs the patient of the benefits and risks of all treatment options [42]. In the ‘shared decision-making model’ the doctor and patient discuss their own treatment preferences and decision-making is shared. In the ‘informed decision-making model’, in contrast, the decision-making process is the sole responsibility of the patient. Both models require that all the relevant treatment options and associated risks and benefits have been explained to the patient. However, findings from older patients’ interviews from three northern European countries reveal that this idealized situation does not always reflect the reality of patients’ experiences.

### Declaration of contribution of authors

NE designed and carried out the secondary analysis and drafted the manuscript. HRWP contributed to the study design, analysis and helped to draft the manuscript. SAP, JS, SP, RD, BDOP contributed to the conception of the study, the gathering of data and helped draft the manuscript. All authors read and approved the final manuscript.

### Collaborators

Van den Block Lieve^a^, Meeussen Koen^a^, Brearley Sarah^e^, Caraceni Augusto^g^, Cohen Joachim^a^, Costantini Massimo^h^, Francke Anneke^b^, Harding Richard^c,d^, Higginson Irene J^c,d^, Kaasa Stein^f^, Linden Karen^k^ , Miccinesi Guido^i^, Onwuteaka-Philipsen Bregje^b^, Pardon Koen^a^, Pasman Roeline^b^, Pautex Sophie^j^, Payne Sheila^e^, Deliens Luc^a,b^

## Competing interests

The authors declare no competing interest.

## Pre-publication history

The pre-publication history for this paper can be accessed here:

http://www.biomedcentral.com/1472-684X/11/24/prepub
